# Predictors of caregiver adherence to administration of amodiaquine during delivery of seasonal malaria chemoprevention in Nigeria, Burkina Faso, Chad, and Togo

**DOI:** 10.1186/s12936-023-04576-5

**Published:** 2023-05-05

**Authors:** Taiwo Ibinaiye, Olusola Oresanya, Chibuzo Oguoma, Adaeze Aidenagbon, Olabisi Ogunmola, Christian Rassi, Sol Richardson

**Affiliations:** 1 Malaria Consortium Nigeria, 33 Pope John Paul Street, Maitama, Abuja-FCT, Nigeria; 2 Malaria Consortium UK, The Green House, 244-254 Cambridge Heath Road, London, E2 9DA UK; 3 Vanke School of Public Health, Tsinghua University, Beijing, 100083 China

**Keywords:** Malaria, Seasonal malaria chemoprevention, Community engagement, Amodiaquine

## Abstract

**Background:**

Malaria is the leading cause of morbidity and mortality among infants and children under-five in sub-Saharan Africa. In the Sahel, seasonal malaria chemoprevention (SMC) is delivered door-to-door in monthly cycles. In each cycle, children are administered sulfadoxine–pyrimethamine (SP) plus amodiaquine (AQ) on Day 1 by community distributors, and AQ on Day 2 and Day 3 by caregivers. Non-adherence to AQ administration by caregivers has implications for emergence of antimalarial resistance.

**Methods:**

Predictors of non-adherence to administration of AQ on Day 2 and Day 3 among caregivers of children aged 3–59 months who had received Day 1 SP and AQ during the last 2020 SMC cycle (n = 12,730) were analysed using data from SMC coverage surveys in Nigeria, Burkina Faso and Togo, and fitting multivariate random-effects logistic regression models.

**Results:**

Previous adverse reaction to SMC medicines by eligible children (OR: 0.29, 95% CI 0.24–0.36, p < 0.001), awareness of the importance of administering Day 2 and Day 3 AQ (OR: 2.19, 95% CI 1.69–2.82, p < 0.001), caregiver age, and home visits to caregivers delivered by the Lead Mothers intervention in Nigeria (OR: 2.50, 95% CI 1.93–2.24, p < 0.001), were significantly associated with caregiver adherence to Day 2 and Day 3 AQ administration.

**Conclusions:**

Increasing caregivers’ knowledge of SMC and interventions such as Lead Mothers have the potential to improve full adherence to AQ administration.

## Background

Malaria has been identified as the leading cause of morbidity and mortality among infants and children under-five in sub-Saharan Africa [1]. In the Sahel region, most of the disease burden occurs during a distinct high-transmission period corresponding to the rainy season. In these areas, seasonal malaria chemoprevention (SMC) using sulfadoxine–pyrimethamine (SP) plus amodiaquine (AQ) is delivered as a community-based intervention involving annual SMC rounds comprising four or five monthly cycles [2]; SMC was endorsed by the World Health Organization (WHO) in 2012 as a cost-effective tool for malaria prevention in children aged 3–59 months [3], which has been associated with a significant reduction in malaria cases [4], malaria morbidity [5] and malaria deaths, with high effectiveness when implemented at scale [6]. SMC has since been implemented in thirteen countries in the Sahel region of Africa and over 45 million eligible children were reached in 2021 [7].

Each full monthly course of SMC medicines consists of one dose each of SP and AQ (SPAQ) on the first day (Day 1) administered by caregivers under the guidance of SMC community distributors, while daily doses of AQ are administered by caregivers on Day 2 and 3. While full adherence to the SMC course by eligible children is essential to ensuring full chemopreventive effectiveness is achieved [8], development of resistance to SP and AQ among circulating *Plasmodium falciparum* parasites has been identified as a major concern for the ongoing viability of SMC programmes [9]. Non-adherence to the full course of SP and AQ has implications for potential acceleration of emergence of resistance to AQ in populations where children are receiving SMC [10–13].

Previous work has shown that caregivers’ socioeconomic position and demographic characteristics, community-level factors, and seasonal or environmental influences, are potential predictors of caregivers’ full adherence to SMC administration in Burkina Faso and Ghana [10, 12, 14]. In a study conducted in Senegal [15], Cissé and Sokhna identified parental socioeconomic background and perceived knowledge of potential adverse events arising from administration of AQ on Day 2 and Day 3 as part of SMC as predictors of non-adherence to full the full course of SP and AQ among children aged under 10 years. The study also found that caregivers who lacked good knowledge of benefits of SMC were less likely to adhere fully to administration of all AQ doses.

### Interventions promoting full adherence to SMC

While community engagement activities common to all SMC programmes disseminate basic messages on SMC via SMC distributors during household visits, in addition to community leaders, town announcers, radio jingles and banners, targeted interventions have also been designed to promote caregivers’ full adherence to administration of all AQ doses. In Nigeria, the ‘lead mothers’ intervention is delivered by female aged 18 years and above with children, who are residents of the community where SMC is implemented and recruited to visit caregivers in their homes following home visits by SMC distributors on Day 1. Their tasks include supporting caregivers in administering Day 2 and Day 3 doses of AQ and disseminating health messages. Outside SMC, the lead mothers intervention has previously been employed to support nutrition projects in Nigeria with successful outcomes [16]. As of 2022, Malaria Consortium has also been trialing the role model intervention in Burkina Faso, Chad, and Togo with a view to evaluating its impacts on SMC outcomes including Day 2 and Day 3 AQ administration [17].

### Study rationale and aim

Although several recent studies have addressed the consequences of partial administration of SMC in a range of sub-Saharan African countries, these mainly focused on considerations of cost-effectiveness and coverage of SMC treatment rather than addressing factors that prevent or discourage full administration of SMC among caregivers [10, 12–15].

Based on the need to identify caregivers most likely to not completely adhere to the full course of SP and AQ and inform potential interventions or modifications to community engagement activities during SMC campaigns, a study based on secondary data analysis was conducted to identify factors predicting adherence to administration of AQ on Day 2 and Day 3 following receipt of SPAQ on Day 1 in four Sahelian countries using data obtained from annual SMC coverage surveys commissioned by Malaria Consortium. As a secondary objective, the association between lead mother visits in Nigeria and adherence was assessed.

## Methods

### Survey methods

Data were obtained from end-of-round SMC coverage surveys conducted in Burkina Faso, Chad, Nigeria and Togo by independent investigators in October–December 2020 after the end of the annual SMC round, described in greater detail elsewhere [18]. Surveys, which took place between October and December 2020 were intended to achieve a representative sample of the target population covered by SMC programmes supported by Malaria Consortium at the country level, or state level in Nigeria. In all four countries, settlements were selected at random with probability proportional to population size to achieve a self-weighting sample. In Nigeria a modified cluster sampling design was employed to select 1320 households each in the states of Bauchi, Jigawa, Kano, Katsina, Kebbi, Sokoto and Yobe in Local Government Areas (LGAs) where SMC was delivered. Only households with at least one child aged 3–59 months were eligible for inclusion in end-of-round surveys. Data were collected by pairs of data collectors using SurveyCTO version 2.70 on mobile devices. In each household a roster was taken of all children aged under 10 years; one eligible child was selected automatically at random by the SurveyCTO application from the roster. Children were considered eligible for receipt of Day 1 SPAQ in cycle 4 if they were aged 3–59 months at the time of SMC distributor visits in that cycle, or if aged over 59 months, they were aged 59 months or less at the beginning of the SMC round in cycle 1 (ascertained by asking caregivers if the child was born after June 2015). Children ineligible for other reasons were not included in our sample, for example those with a known allergy to SP or AQ or with fever or at the time of SMC distributor visits. The analytic sample comprised primary caregivers of randomly-selected eligible children who had received Day 1 SPAQ in cycle 4 of 2020 in the four countries [18].

### Variable definitions

The outcome measure, full caregiver adherence to the SMC regimen, was defined as administration of both the Day 2 and Day 3 AQ treatments to an eligible child; this was operationalised as a binary variable. Variables considered as possible predictors of adherence to Day 2 and Day 3 AQ administration included variables related to socio-demographic characteristics of caregivers, and their awareness and knowledge of SMC. Covariates considered for inclusion in the regression model included lead mother visit during cycle 4 (Nigeria only, yes/no), child age (1 year age bands from age 0 to 4 years), caregiver literacy status (based on caregiver self-reports of being able read and write and any language, yes/no), caregiver employment status (unemployed, retired or out of the workforce/paid or unpaid agricultural work/paid or unpaid manual work, skilled manual or service work/clerical, technical, professional and managerial work), caregiver awareness of SMC (yes/no), caregiver knowledge of the purpose of SMC (yes/no), and knowledge of the reason for administration of Day 2 and Day 3 AQ (yes/no). Caregivers were considered to know the purpose of SMC if they spontaneously responded that SMC is intended to protect children from malaria (or similar response). Caregivers were considered to have knowledge of the reason for administration of Day 2 and Day 3 AQ if they spontaneously responded that it is important for children to receive all doses to ensure full protection against malaria (or similar response).

### Statistical analysis

To identify predictors of caregiver adherence to Day 2 and Day 3 AQ administration, random-effects logistic regression models for binary outcomes were fitted with random intercepts for district (or LGA in Nigeria) to account for geographic clustering in responses for the dependent variable. Post-sampling weights were applied for Nigeria to account for the fact that all states had an equal target sample size regardless of the population of eligible children targeted for SMC delivery. Variables were selected for inclusion in the model using a forward stepwise regression approach based on Collett’s method [19]. In brief, variables were sequentially added to the model and regressed on the dependent variable; they were retained in the model if the likelihood ratio test indicated that their inclusion improved model fit (p < 0.05). Once no additional variable improved model fit the process was stopped with no further variables added.

### Ethical approval

Ethical approval for the surveys was granted by the relevant agencies in each country, including the National Health Research Ethics Committee in Nigeria, the Comité National d'Éthique pour la Recherche en Santé (National Committee for Ethics for Health Research) in Burkina Faso, the Comité National de Bioéthique du Tchad (the National Bioethics Committee of Chad) in Chad, and the Comité Consultatif National de Bioéthique (National Bioethics Committee) in Togo.

## Results

**Fig. 1:**
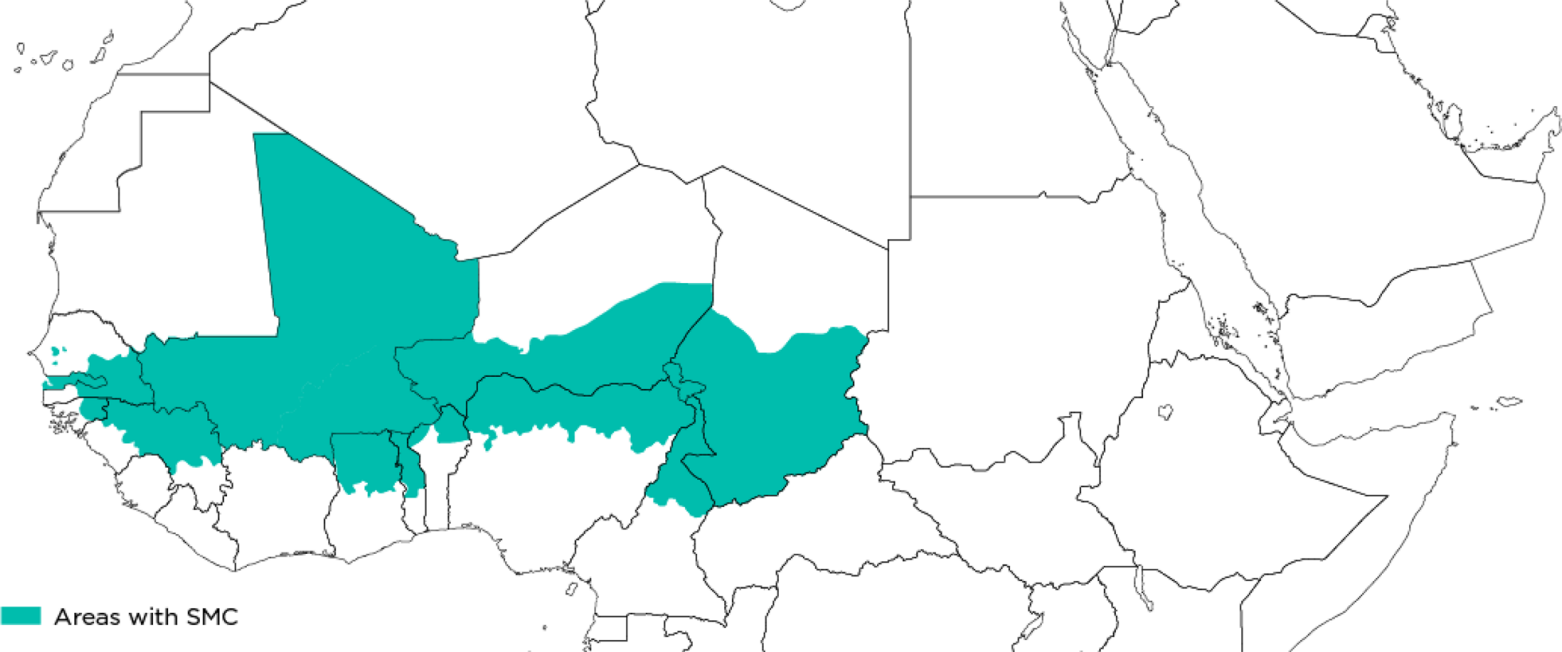
Map of areas with delivery of SMC, North-West Africa, 2020. Green denotes areas, including provinces (Burkina Faso), regions (Chad and Togo) and states (Nigeria), with delivery of SMC during 2020

Figure 1 shows areas of North-West Africa, including states and regions of Nigeria, Burkina Faso, Chad and Togo, where SMC delivered in 2020. The final analytic sample included data from 12,730 caregivers of eligible children from across the four countries without missing observations for any of the variables selected by the forward stepwise procedure. The characteristics of the sample of caregivers and eligible children are shown in Table 1.

**Table 1:** Sample characteristics for end-of-round surveys in Nigeria, Burkina Faso, Chad and Togo (n = 12,730)

Variables and categories	Burkina Faso		Nigeria		Chad		Togo		Total	
	n	%	n	%	n	%	n	%	n	%
Caregiver gender										
Female	2007	93.0	5693	89.2	1697	73.2	1466	78.3	1.863	85.3
Male	150	7.0	689	10.8	621	26.8	407	21.7	1.867	14.7
Total	2157	100.0	6382	100.0	2318	100.0	1873	100.0	12730	100.0
Caregiver age										
< 20 years	117	5.4	374	5.9	186	8.0	61	3.3	738	5.8
20–29 years	995	46.1	2649	41.5	910	39.3	722	38.5	5276	41.4
30–39 years	752	34.9	2250	35.3	825	35.6	765	40.8	4592	36.1
40–49 years	198	9.2	816	12.8	303	13.1	226	12.1	1543	12.1
50–59 years	60	2.8	221	3.5	83	3.6	78	4.2	442	3.5
> 60 years	35	1.6	72	1.1	11	0.5	21	1.1	139	1.1
Total	2157	100.0	6382	100.0	2318	100.0	1873	100.0	1.730	100.0
Caregiver level of education										
No education	1376	63.8	2102	32.9	1217	52.5	805	43.0	5500	43.2
Informal or religious education	61	2.8	2432	38.1	344	14.8	26	1.4	2863	22.5
Primary school	316	14.6	684	10.7	282	12.2	459	24.5	1741	13.7
Secondary school	379	17.6	882	13.8	353	15.2	515	27.5	2129	16.7
Higher education	25	1.2	282	4.4	122	5.3	68	3.6	497	3.9
Total	2157	100.0	6382	100.0	2318	100.0	1873	100.0	12.730	100.0

Caregiver occupation										
Unemployed	645	29.9	2536	39.7	1447	62.4	403	21.5	5031	39.5
Agriculture	1042	48.3	559	8.8	534	23.0	859	45.9	2994	23.5
Unskilled manual work	57	2.6	703	11.0	46	2.0	85	4.5	891	7.0
Sales services and Skilled manual	351	16.3	2409	37.7	237	10.2	474	25.3	3471	27.3
Clerical, technical, professional or managerial	62	2.9	175	2.7	54	2.3	52	2.8	343	2.7
Total	2157	100.0	6382	100.0	2318	100.0	1873	100.0	12730	100.0
Caregiver self-reported literacy										
No	1381	64.0	3241	50.8	1280	55.2	816	43.6	6718	52.8
Yes	776	36.0	3141	49.2	1038	44.8	1057	56.4	6012	47.2
Total	2157	100.0	6382	100.0	2318	100.0	1.873	100.0	12730	100.0
Day 2 and 3 AQ administration (cycle 4)										
No	26	1.2	352	5.5	138	6.0	49	2.6	565	4.4
Yes	2131	98.8	6030	94.5	2180	94.0	1824	97.4	12165	95.6
Total	2157	100.0	6382	100.0	2318	100.0	1873	100.0	12730	100.0

The overall the proportion of caregivers from all four countries combined that reported administering AQ on Day 2 and Day 3 to the randomly-selected child was 95.6%, ranging from 94.0% in Togo to 98.8% in Burkina Faso. The majority of primary caregivers surveyed were female, ranging from 73.2% in Chad to 93.0% in Burkina Faso.

Table 2 presents the results of the multiple logistic regression model with variables selected by forward stepwise selection. Odds ratios, 95% confidence intervals (95% CI) and p-values are provided for each selected variable.

**Table 2:** Results of logistic regression model of variables predicting adherence to Day 2 and Day 3 SMC administration by caregivers of eligible children in Nigeria, Burkina Faso, Chad and Togo (n = 12.730)

Variable	Category	Odds ratio	p	95% CI	
Lead mother visit	No	Ref			
	Yes	2.500	< 0.001	1.930	3.237
Caregiver self-reported literacy	No	Ref			
	Yes	0.950	0.707	0.726	1.242
Heard date of cycle 4 SMC delivery	No	Ref			Ref
	Yes	0.501	< 0.001	0.367	0.685
Adverse reactions to SMC medicines	No	Ref			
	Yes	0.293	< 0.001	0.237	0.362
Caregiver awareness of importance of AQ administration	No	Ref			
	Yes	2.190	< 0.001	1.697	2.825
Caregiver awareness of purpose of SMC	No	Ref			
	Yes	1.397	0.009	1.088	1.793
Caregiver awareness of SMC	No	Ref			
	Yes	1.377	0.005	1.104	1.717
Caregiver age	< 20 years	Ref			
	20–29 years	0.559	0.282	0.194	1.611
	30–39 years	0.570	0.299	0.197	1.646
	40–49 years	0.393	0.090	0.133	1.158
	50–59 years	0.307	0.044	0.097	0.966
	> 60 years	0.410	0.215	0.100	1.680
Caregiver level of education	No education	Ref			
	Informal or religious education	1.021	0.882	0.779	1.338
	Primary school	1.028	0.882	0.715	1.479
	Junior secondary school	1.083	0.707	0.715	1.638
	Senior secondary school	0.832	0.442	0.520	1.331
	Higher education	1.237	0.474	0.691	2.213
Age of selected child	3–11 months	Ref			
	1 years	1.428	0.099	0.935	2.182
	2 years	1.420	0.094	0.942	2.139
	3 years	1.442	0.078	0.959	2.168
	4 years	1.311	0.184	0.880	1.953

The results showed that an adverse reaction to SMC medicines by the randomly-selected child was negatively associated with lower odds of adherence to Day 2 and Da y3 AQ administration (OR: 0.29, 95% CI 0.24–0.36, p < 0.001). Meanwhile, awareness of SMC (OR: 1.37, 95% CI 1.10–1.72, p < 0.001), awareness of the importance of administering Day 2 and Day 3 AQ (OR: 2.19, 95% CI 1.69–2.82, p < 0.001) and knowledge of the purpose of SMC (OR: 1.40, 95% CI 1.09–1.79, p = 0.009) were all significantly and positively associated with greater odds of adherence to Day 2 and Day 3 AQ administration. In addition, we found a weak association between age of caregiver and odds of adherence to Day 2 and Day 3 AQ administration; the model results indicated that compared with caregivers aged under 20 years, those aged 40–49 years (OR: 0.39, 95% CI 0.13–1.16, p = 0.090) and 50–59 years (OR: 0.31, 95% CI 0.10–0.97, p = 0.044) had a lower odds of administering Day 2 and Day 3 AQ. A visit by a lead mother following a home visit by a SMC distributor in cycle 4 (Nigeria only) was also positively and significantly associated with higher odds of adherence to Day 2 and Day 3 AQ administration by caregivers (OR: 2.50, 95% CI 1.93–2.24, p < 0.001).

## Discussion

In summary, the results of the regression models found that lead mother visits, adverse reactions to SMC medicines, and awareness of SMC and knowledge of the importance of AQ administration were predictors of full adherence to administration of Day 2 and Day 3 AQ among caregivers of eligible children in the four countries surveyed. These results imply that the more the level of knowledge of caregivers about the benefits of SMC AQ to their children in general, the more likely their children receive Day 2 and Day 3 AQ. These findings corroborate those of Sokhna (2016) that knowledge of SMC benefits was one of the determinants of caregiver adherence to administration AQ for children aged under-10 years. Unlike Diawara et al*.* [10], Druetz et al*.* [14], and Chatio et al*.* [12] who found socioeconomic and community-level factors influenced full AQ administration of among caregivers in Burkina Faso and Ghana, the variable selection procedure did not find that inclusion of these variables improved model goodness-of-fit or were not available in the end-of-round coverage survey datasets, and these were not included in the final model.

Lower odds of Day 2 and Day 3 AQ administration were also found among caregivers whose eligible children experienced adverse reactions to SMC medicines. It is arguable that caregivers whose children had previously experienced one form of adverse reaction following administration of Day 1 SPAQ are less likely to adhere to Day 2 and Day 3 administration of AQ due to concerns that the same adverse reactions might occur. The study’s findings are consistent with Cissé and Sokhna [15], who argued that perceived knowledge of complications, or perceived risk arising from uptake of SMC influenced odds of adherence to complete administration of AQ for under-10 children among caregivers in Senegal.

Lead mother visits in cycle 4 were also positively associated with adherence to Day 2 and Day 3 AQ administration. While this finding is expected due to the content of lead mothers’ messaging including AQ administration, the large effect size (OR: 2.5) found in the analysis points to the effectiveness of the intervention in promoting caregivers’ full adherence to SMC.

## Strengths and limitations

Strengths of this study include its use of independent surveys conducted by external investigators not affiliated with SMC programmes, its large analytic sample, and its inclusion of multiple countries allowing generalizability of its results. This study illustrates the potential of routine coverage surveys with inclusion of household sociodemographic variables, which yield representative samples of eligible children, caregivers and households with eligible children, to provide data to address research questions on SMC and inform future improvements. Its limitations include reliance on self-reporting by caregivers, particularly for variables such as caregiver literacy which may have been subject to social desirability bias, and the time delay of up to 10 weeks between delivery of SP and AQ in cycle 4 and surveys which may have reduced reliability of reporting and resulted in potential for recall bias. In addition, the question on adverse reactions to SMC medicines among of children who received Day 1 SPAQ was not specific to any of the four doses of SP or AQ, and inclusion of a relevant question would have made it possible to ascertain whether the adverse reaction started before either of the Day 2 or Day 3 AQ doses.

## Conclusions and policy recommendations

The study’s finding that knowledge of SMC was one of key the factors that determined caregivers’ adherence to Day 2 and Day 3 administration of AQ to children within 3–59 months in West Africa implies that additional efforts should be made to strengthen caregivers’ awareness and knowledge of increase awareness of SMC. In addition to increasing the intensity of standard community engagement, for example through radio jingles, banners and meetings with community and religious leaders, these efforts should also ensure that messages they deliver are effective at informing caregivers of the importance of, and reason for, Day 2 and Day 3 AQ administration. While there was a weak association between caregiver age and adherence to Day 2 and Day AQ administration, it may be suggested that sensitization efforts tailor their choice of medium and messaging to ensure caregivers of all ages are reached. Messages may also seek to allay caregivers’ concerns around Day 2 and Day 3 AQ administration if their children should experience minor adverse effects following Day 1 administration of SP and AQ.

Lead mothers may also play a key role in ensuring full adherence to AQ administration among caregivers, as evidenced by our results. While similar interventions may be tailored to other countries, attention should also be paid to the content of messaging to delivered to caregivers by lead mothers. Malaria Consortium is conducting a study in Minjibir LGA in Kano State, Nigeria, to evaluate impacts of existing and enhanced version of the lead mothers intervention, and will assess whether enhancements are associated with additional improvements in caregiver adherence to Day 2 and Day 3 AQ administration [20].

## Data Availability

Data employed in this study are available from the authors upon reasonable request.
